# Establishment and Early Days of the Medical Department of the University of Buffalo1Read at the seventy-fifth anniversary of the Medical Society of the County of Erie, January 14, 1896.

**Published:** 1896-05

**Authors:** C. C. Wyckoff

**Affiliations:** Buffalo, N. Y.; 482 Delaware Avenue


### ESTABLISHMENT AND EARLY DAYS OF THE MEDICAL DEPARTMENT OF THE UNIVERSITY OF BUFFALO.[1]

By C. C. WYCKOFF, M. D., Buffalo, N. Y.

THE attention of members of the medical profession, not only in this immediate neighborhood, but at a distance, had been frequently directed to this city as a point affording peculiar advantages for medical instruction. The city had in 1845 attained a population of about 30,000 and, by men of good judgment, it was confidently predicted that its population would be doubled in ten years. There was every reason to suppose that Buffalo would maintain its position as the commercial emporium of the northwest. Taking into view its prospective importance and the proportionate facilities and materials for medical instruction, the extensive territory with which it was connected by means of the various channels of communication concentrating here from every direction, as to a central point, it was thought that there was no place between the extreme east and the farthest extremity of our lakes that possessed equal advantages as a locality for a school of medicine with this.

During the legislative session of 1846 an act was passed incorporating the University of Buffalo. This act was broad and comprehensive in its character, providing not only for a collegiate institution of the highest grade, but for a university with a complete organisation of law, medical and theological departments. By the provisions of the act the commissioners named in it were empowered to organise a university whenever $20,000 had been

1. Read at the seventy-fifth anniversary of the Medical Society of the County of Erie, January 14, 1896.

subscribed to its capital stock. This amount having been subscribed, the commissioners gave public notice of the fact, and advertised that a meeting of the stockholders for the election of members of the council, who should administer the affairs of the University, would be held on the 22d day of May, 1846. The stockholders met on the day appointed and the following-named gentlemen were elected to constitute the first council of the University of Buffalo, dividing themselves into four classes to serve one, two, three and four years each: Ira A. Blossom, Isaac Sherman, Theodotus Burwell, James O. Putnam, Gaius B. Rich, William A. Bird, George R. Babcock, Hiram A. Tucker, Joseph G. Masten, Thomas M. Foote, John D. Shepard, Millard Fillmore, Elbridge G. Spaulding, Orson Phelps, Orsamus H. Marshall, George W. Clinton.

On the evening of May 25th this council held the first meeting and it was decided to organise the medical department forthwith, which was done by establishing seven professorships and the election of the following-named gentlemen: James Hadley, M. D., professor of chemistry and pharmacy; Charles B. Coventry, M. D., physiology and medical jurisprudence; James Webster, M. D., general and special anatomy; Charles A. Lee, M. D., pathology and materia medica; Frank H. Hamilton, M. D., principles and practice of surgery and clinical surgery; James P. White, M. D., obstetrics and diseases of women and children; Austin Flint, M. D., principles and practice of medicine and clinical medicine. Five of these seven chairs were occupied by incumbents of similar positions in Geneva medical college. These five professors—namely, Hadley, Coventry, Webster, Lee and Hamilton—still retained their connection with Geneva medical college, and hence for themselves and the students who might desire to attend lectures both in Buffalo and Geneva, it was decided that the first course should be a spring course. It commenced on the 24th day of February, 1847, and continued the usual period of sixteen weeks; the lectures in every department being as full as the course in Geneva.

All these seven professors, especially those from the faculty of Geneva, were men eminent in their several departments and in the profession at large. This at once placed the medical department of the University of Buffalo among the first medical colleges of this country. The other two professors proved themselves worthy compeers and fulfilled the duties devolving upon them with eminent satisfaction. It is proper to state, however, that Professor George Hadley delivered the lectures on chemistry in place of his father, Professor James Hadley, and was appointed to fill the place permanently and fully equaled his father as a teacher.

Corydon La Ford, M. D., who afterward became one of the most famous teachers of anatomy in this country, was appointed demonstrator of anatomy. He was untiring in his efforts as a teacher, and if all the students did not become good anatomists under his teaching and that of Professor Webster it was entirely due to their own indolence or inattention. Clinical instruction was not neglected during the first term of lectures. The Buffalo Dispensary and the Medical College Dispensary furnished a large number of cases—surgical under the management of Professor Hamilton and medical under Professor Austin Flint.

I wish I could present to you a graphic picture of these first seven professors as they appear to me in memory—the dignified and serious Hadleys, father and son; the courtly Christian gentleman, Professor Coventry, whose innate modesty put him to the blush upon demonstrating his obstetrical lectures upon the manikin; the agile and oftentimes brilliant Hamilton, entering the amphitheater almost upon a run, lecturing as he came, and seeming only desirous of improving every moment to give us the benefit of his vast store of learning; the more dignified Flint, who at the beginning of his career as a lecturer was somewhat inclined to verboseness, but who afterward attained an eminence in this branch of the profession as may make us justly proud of having given him to the world; the daring White, who raised such a storm of abuse, which he manfully met, when he introduced "demonstrative midwifery;" the companionable, convivial Webster, his own worst enemy, who was masterly at dissection, lecturing as rapidly as the scalpel cut into the tissues of the subject, never for a moment at a loss for words to explain the hidden course of nature. Oftentimes Dr. La Ford would have to perform the duties of a lecturer as well as those of a demonstrator of anatomy, but it was at no loss to the students. Professor Lee was perhaps less known to us, as he always retained his home in New York, but his uniform kindness made him popular, although his subject was dry and prosy.

The first term of this institution started off with sixty names on the register, which was considered by the faculty as very encouraging, especially as they had adhered to a cash system exclusively, while many colleges of that day were in the habit of extending credit to some of their students for tickets. The council of the University rented for a term of years an edifice located on the corner of Washington and Seneca streets, that had been used, I think, as a church, which after suitable repairs and alterations proved admirably adapted for the purpose of a medical college. A pictorial representation of the college building was correctly given in the first annual announcement.

The *Commercial Advertiser* of Wednesday evening, June 16, 1847, contained this report of the first commencement of the medical college :

The commencement of the Medical Department of the University of Buffalo was held this forenoon at the First Presbyterian Church, according to the published program of exercises. After an earnest and appropriate prayer by the Rev. A. T. Hopkins, the Hon. Millard Fillmore, chancellor of the university, delivered a brief address, reviewing the educational history of our city, and drawing from the experiences of the past and the facts of the present, incentives to persevere in the efforts to build up at this point a university that should be commensurate with the present and future wants of our city, and alike honorable to its enterprise and zeal for intellectual culture.

The degree of doctor of medicine was then conferred by the chancellor upon the following young gentlemen : Bela E. Phelps, George B. Parker, S. C. Rogers, Jas. E. King, M. H. Andrews, John P. Dudley, Delos M. Norton, H. D. Garvin, Sidney A. Foss, Wells Taber, Horace S. Lindsley, J. A. Whiting, H. W. Barrett, John Hardy, George Abbott, William Ring, Z. A. Blake.

An address to the graduates, characterised by good taste and replete with excellent advice, was delivered by the dean of the medical faculty, Professor F. H. Hamilton, when, after singing by the choir, the audience was dismissed with a benediction by the Rev. Mr. Schuyler, of St. John's church.

Thus closed the first literary exercises of the kind ever witnessed in Buffalo. The church was thronged by a large attendance of our most estimable citizens, and from the happy manner in which everything went off, and the general interest apparently felt in the prosperity of the institution, we anticipate the best results for the future.

The number of students graduated at the first commencement was seventeen. At the second commencement, which took place June 14, 1848, there were thirty-two graduates, about doubling the number of the preceding year, while the number of students enrolled for this year was ninety-five.

At this commencement, at which I received my degree as M. D.,

Dr. Thomas M. Foote conferred the degrees in the absence of the chancellor, Hon. Millard Fillmore, and Professor Austin Flint delivered the address to the graduates.

Land had been purchased on the corner of Main and Virginia streets for a medical college building, which was then in process of construction.

The public-spirited citizens who contributed towards the construction of this first permanent home for the medical department of the University of Buffalo were A. D. Patchin, whose name heads the list with a subscription of $500. The largest donor was Jesse Ketchum, who gave $600. Subscriptions of $200 each were given by Albert H. Tracy, George W. Tifft, E. G. Spaulding and Jabez Goodell. Eighty subscriptions of $100 each were given and the remainder made up in smaller amounts, until an aggregate of $12,000 was reached, which with an appropriation of $2,000 from the state assured a sufficient sum to warrant the construction of the then new, but now old medical college building of the University of Buffalo, so well known to all of its alumni. This building, which at that time was considered a very fine structure, as compared with the handsome edifice which now shelters the University of Buffalo, proves the original founders were not only true prophets of the prospective growth of our city, but also that the medical profession has kept pace with that growth in the advance of science as well as in prosperity.

482 Delaware Avenue.

